# “Pure” severe aortic stenosis without concomitant valvular heart diseases: echocardiographic and pathophysiological features

**DOI:** 10.1007/s10554-020-01907-4

**Published:** 2020-06-04

**Authors:** J. Kandels, B. Tayal, A. Hagendorff, D. Lavall, U. Laufs, P. Sogaard, N. H. Andersen, S. Stöbe

**Affiliations:** 1 Department of Cardiology, University Hospital Leipzig, Liebigstraße 20, 04103 Leipzig, Germany; 2 Department of Cardiology, University Hospital Aalborg, Hobrovej 18-22, 9100 Aalborg, Denmark

**Keywords:** Transthoracic echocardiography, Severe aortic valve stenosis, Left ventricular hypertrophy, Diastolic dysfunction, Pulmonary hypertension

## Abstract

**Purpose:**

In echocardiography the severity of aortic stenosis (AS) is defined by effective orifice area (EOA), mean pressure gradient (mPG_AV_) and transvalvular flow velocity (maxV_AV_). The hypothesis of the present study was to confirm the pathophysiological presence of combined left ventricular hypertrophy (LVH), diastolic dysfunction (DD) and pulmonary artery hypertension (PAH) in patients with “pure” severe AS.

**Methods and Results:**

Patients (n = 306) with asymptomatic (n = 133) and symptomatic (n = 173) “pure” severe AS (mean age 78 ± 9.5 years) defined by indexed EOA < 0.6 cm^2^ were enrolled between 2014 and 2016. AS patients were divided into 4 subgroups according to mPG_AV_ and indexed left ventricular stroke volume: low flow (LF) low gradient (LG)-AS (n = 133), normal flow (NF) LG-AS (n = 91), LF high gradient (HG)-AS (n = 21) and NFHG-AS (n = 61). Patients with “pure” severe AS showed mean mPG_AV_ of 31.7 ± 9.1 mmHg and mean maxV_AV_ of 3.8 ± 0.6 m/s. Only 131 of 306 patients (43%) exhibited mPG_AV_ > 40 mmHg and maxV_AV_ > 4 m/s documenting incongruencies of the AS severity assessment by Doppler echocardiography. LVH was documented in 81%, DD in 76% and PAH in 80% of AS patients. 54% of “pure” AS patients exhibited all three alterations. Ranges of mPG_AV_ and maxV_AV_ were higher in patients with all three alterations compared to patients with less than three. 224 (73%) patients presented LG-conditions and 82 (27%) HG-conditions. LVH was predominant in NF-AS (p = 0.014) and PAH in LFHG-AS (p = 0.014). Patients’ treatment was retrospectively assessed (surgery: n = 100, TAVI: n = 48, optimal medical treatment: n = 156).

**Conclusion:**

In patients with “pure” AS according to current guidelines the presence of combined LVH, DD and PAH as accepted pathophysiological sequelae of severe AS cannot be confirmed. Probably, the detection of these secondary cardiac alterations might improve the diagnostic algorithm to avoid overestimation of AS severity.

## Introduction

Aortic valve stenosis (AS) due to degenerative calcifications is the most common valvular heart disease [1]. The prevalence of severe AS increases with age to 3–4% in individuals > 75 years [2]. Recent recommendations for the evaluation of AS by transthoracic echocardiography (TTE) are solely performed by Doppler-derived parameters [3]. Peak transvalvular flow velocity (maxV_AV_), mean transvalvular pressure gradient (mPG_AV_) and effective aortic orifice area (EOA) calculated by the continuity equation are recommended as the primary key parameters to evaluate AS severity. Severe AS is characterized by maxV_AV_ > 4.0 m/s, mPG_AV_ > 40mmHG, EOA < 1cm^2^ (indexed < 0.6 cm^2^/m^2^) and/or the ratio between peak velocity determined at the level of the LV outflow tract (maxV_LVOT_) and maxV_AV_ < 0.25 (maxV_LVOT_/maxV_AV_). However, maxV_AV_, mPG_AV_ and EOA are frequently incongruent in echocardiographic examinations [4, 5]. With respect to the still un-known incidence rate of severe AS the detection of structural and functional cardiac alterations might improve the diagnostic criteria of severe AS. Pathophysiological consequences due to the narrowing of the aortic valve (AV) orifice area, e.g. left ventricular hypertrophy (LVH), diastolic dysfunction (DD) and pulmonary artery hypertension (PAH) are generally assumed in patients with severe AS. Severe AS induces an increase of LV pressure followed by the development of concentric LVH. Concentric LVH leads to a higher diastolic pressure–volume relationship resulting in an increased LV end-diastolic pressure (LVEDP) as evidence of DD. Pulmonary vascular resistance increases with progression of DD indicated by an increase of systolic pulmonary artery pressure (sPAP) [6–10]. LVH, DD and PAH are obviously cardiovascular alterations due to severe AS, which are predictive cardiovascular risk factors shown by previous studies [11–14]. However, despite the well-known pathophysiology of severe AS LVH [15], DD [16] and PAH [17, 18] are not observed in all patients with severe AS as reported in the literature. Further, LVH, DD and PAH can be induced by other diseases independently of AS. According to these circumstances it might be possible that either the pathophysiological sequelae of AS are not fully understood or that patients with hemodynamically not relevant AS will also be characterized as severe AS according to current guideline criteria [19].

The aims of the present study were to analyze the discrepancies between echocardiographic parameters in patients with “pure” severe AS defined by current guideline criteria and to analyze the presence of LVH, DD and PAH in these highly selected patients with “pure” severe AS [8–10]. It was hypothesized that “pure” severe AS is correctly characterized by the accepted pathophysiological sequelae with respect to the presence of LVH, DD and PAH irrespectively of AS subtypes (classified by mPG_AV_ and flow conditions).

## Methods

**Fig. 1:**
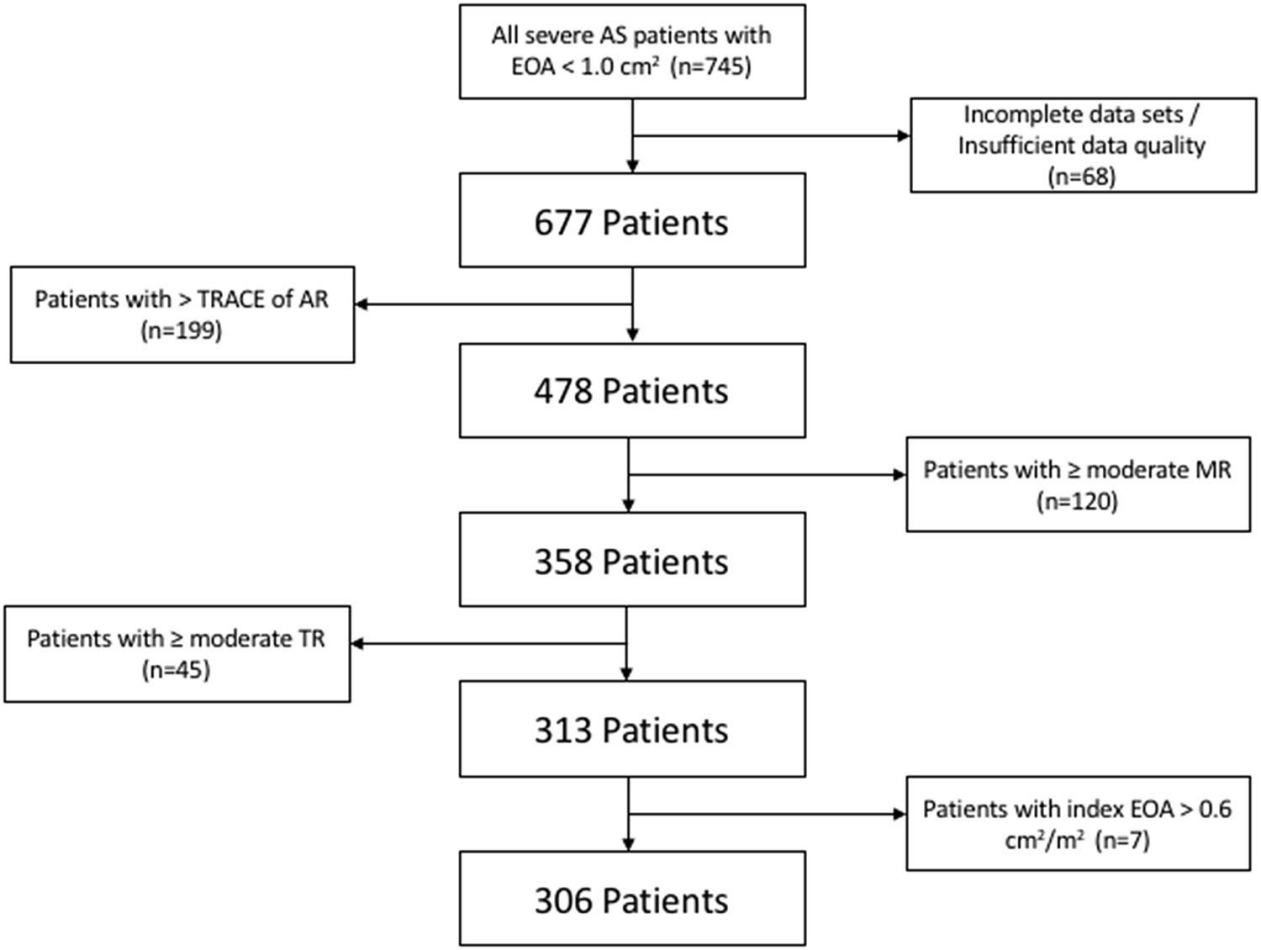
Flow chart of the selection criteria defining “pure” AS patients. AS = Aortic stenosis; EOA = Effective Orifice Area; AR = Aortic valve regurgitation; MR = Mitral regurgitation; TR = Tricuspid regurgitation

In this retrospective study, 745 patients with severe AS defined by an EOA < 1cm^2^ (indexed < 0.6 cm^2^/m^2^), who underwent transthoracic echocardiography (TTE) at the University Hospital Leipzig between January 2014 and December 2016, were analyzed (Fig. 1). Patients with additionally mild to severe aortic regurgitation (AR) and /or concomitant moderate or severe mitral and/or tricuspid valve disease were excluded. Thus, only AS patients with so-called trace AR and mild mitral and/or tricuspid valve disease were enclosed (Fig. 1). Because the former definition of trace AR depends on color-coded Doppler imaging criteria of the nineties [20, 21], trace AR was defined by the following criteria: (1) a pinhead-sized origin of the regurgitation jet, (2) a pressure half time > 750 ms, if continuous-wave (CW) Doppler documented no intercept angle between the ultrasound beam and the direction of blood flow of the regurgitant velocities, and/or (3) a non-holodiastolic AR documented by an anatomical colour-M-Mode. Assessment of mitral and tricuspid valve disease was performed according to current recommendations [10]. Due to the predefined selection criteria the analysis has been performed in a highly selected cohort of so-called “pure” AS patients (n = 306; mean age 78 ± 9.5 years; symptomatic: n = 173; asymptomatic: n = 133), in whom a complete echocardiographic assessment has been performed. The entity of “pure” AS is defined as AS without concomitant valvular heart diseases. However, these “pure” AS patients may have comorbidities, e.g. arterial hypertension, coronary artery disease, diabetes mellitus etc., which obviously do not influence the Doppler echocardiographic assessment of AS severity. The study design was approved by the local ethical committee. Clinical characteristics of the study population were collected from medical records. Patients’ treatment has been retrospectively assessed until December 2019. Surgical valve replacement (n = 100), transcatheter aortic valve implantation (TAVI, n = 48) or optimal medical treatment (OMT) (n = 156) as well as deaths (n = 29) were assessed.

### Classification of severe AS

Patients were grouped according to the current recommendations with respect to mPG_AV_ and indexed LV stroke volume (SVi) [10]. Patients with mPG_AV_ < 40 mmHG were defined as low gradient (LG)-AS and patients with mPG_AV_ ≥ 40 mmHg as high gradient-(HG) AS. Patients with SVi (assessed by Doppler echocardiography) ≤ 35 ml/m^2^ were defined as low flow (LF)-AS and patients with SVi > 35 ml/m^2^ were defined as normal flow (NF)-AS. In total, all patients were divided into four subgroups: LFLG-AS, NFLG-AS, LFHG-AS, NFHG-AS. In addition, patients of AS subgroups were defined as AS patients with normal (LV ejection fraction (EF) ≥ 55%) and reduced LV systolic function (LVEF < 55%) with respect to the proposed grading of AS subgroups [22].

### Basic echocardiographic examination

**Fig. 2:**
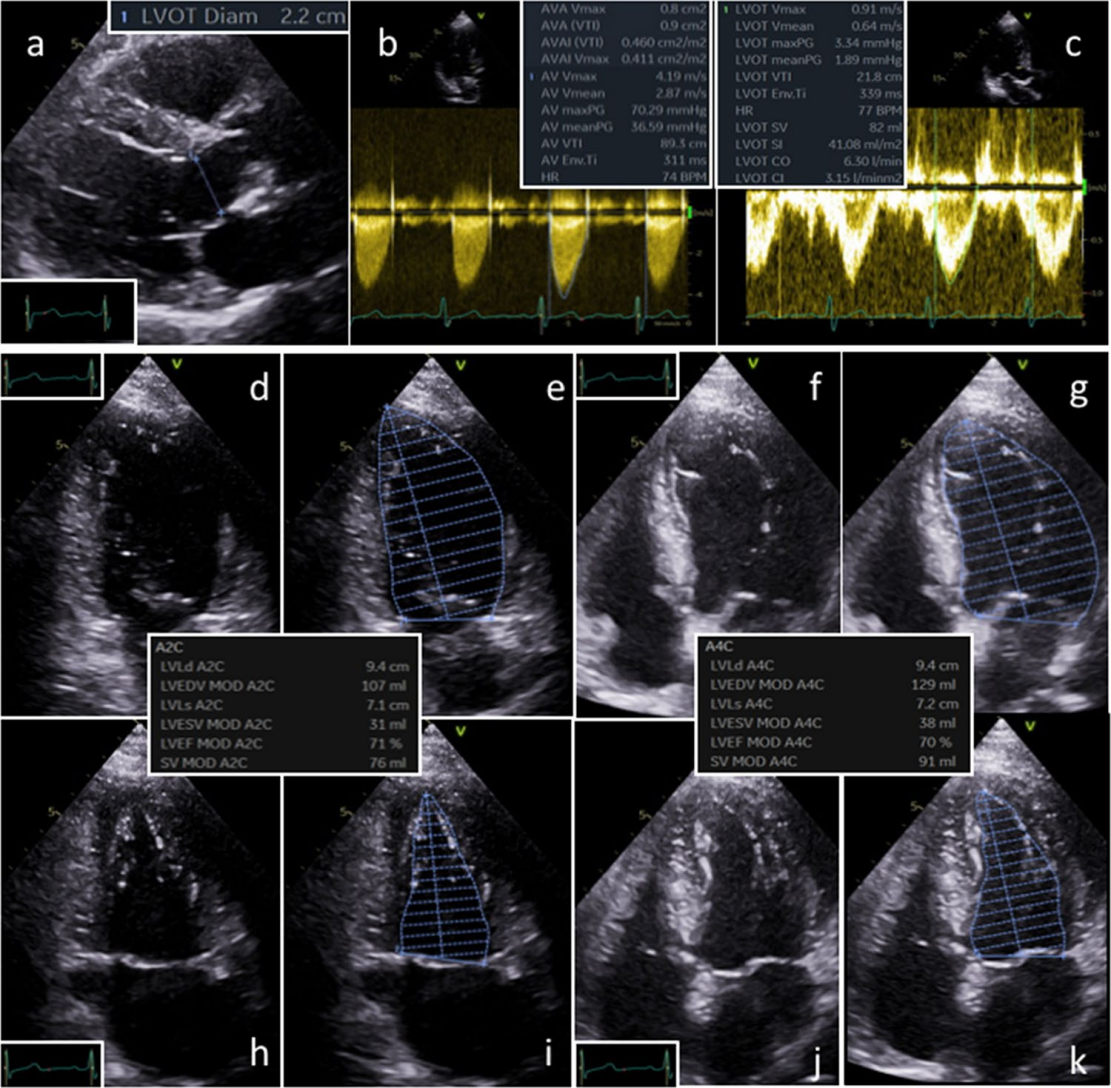
Assessment of effective Aortic orifice Valve Area (AVA) by continuity equation (a-c) and determination of left ventricular stroke volume (LVSV) by Doppler method (a,c) and by Simpson’s method (d-k). LV = left ventricle, LVOT = left ventricular outflow tract, 2C = 2-chamber, 4C = 4-chamber, SV = stroke volume, ESV = end-systolic volume, EDV = end-diastolic volume, EF = ejection fraction, Vmax = maximum flow velocity, maxPG = maximum pressure gradient, meanPG = mean pressure gradient VTI = velocity time integral, CO = cardiac output, HR = heart rate, CI = cardiac index, SI = stroke index

TTE was performed using a Vivid e9 or Vivid e95 ultrasound system with a M5-S phased array probe (GE Healthcare Vingmed Ultrasound AS, Horten, Norway). Echocardiographic analyses were performed with the EchoPac software (Version 202, GE Healthcare Vingmed Ultrasound AS, Horten, Norway). The EOA was calculated by the continuity equation: EOA = (CSA_LVOT_ x VTI_LVOT_)/VTI_AV_ (VTI = velocity time integral). The cross-sectional area of the left ventricular outflow tract (LVOT) (CSA_LVOT_) was calculated by the following equation: CSA_LVOT_ = π x (D_LVOT_/2)^2^. The diameter of the LVOT (D_LVOT_) was determined in the parasternal long axis view (Fig. 2a). The transvalvular VTI (VTI_AV_) was assessed by the continuous wave (CW)-Doppler and the mean transvalvular velocity (meanV_AV_) was assessed to calculate mPG_AV_ applying the simplified Bernoulli equation: mPG_AV_ = 4 x (meanV_AV_)^2^ (Fig. 2b). The pre-stenotic VTI of the LVOT (VTI_LVOT_) was measured by pulsed wave (PW) Doppler in the apical long axis view by positioning the sample volume exactly at D_LVOT_ measurement position (Fig. 2c). The LV stroke volume (SV_LV-Doppler_) was calculated by the following equation using PW Doppler: SV_LV-Doppler_ = CSA_LVOT_ x VTI_LVOT_. LVEF, LV end-diastolic and end-systolic volumes (LVEDV, LVESV) and LVSV_LV-bipl_ (SV_LV-bipl_ = LVEDV–LVESV) were assessed by LV biplane planimetry by the modified Simpson’s rule in the apical 2- and 4-chamber view (Fig. 2d–k). Regarding both approaches SVi was calculated by dividing LVSV by the body surface area (BSA). EOA was also determined by replacing LVSV_LV-Doppler_ with LVSV_LV-bipl_ [8].

### Left ventricular hypertrophy

**Fig. 3:**
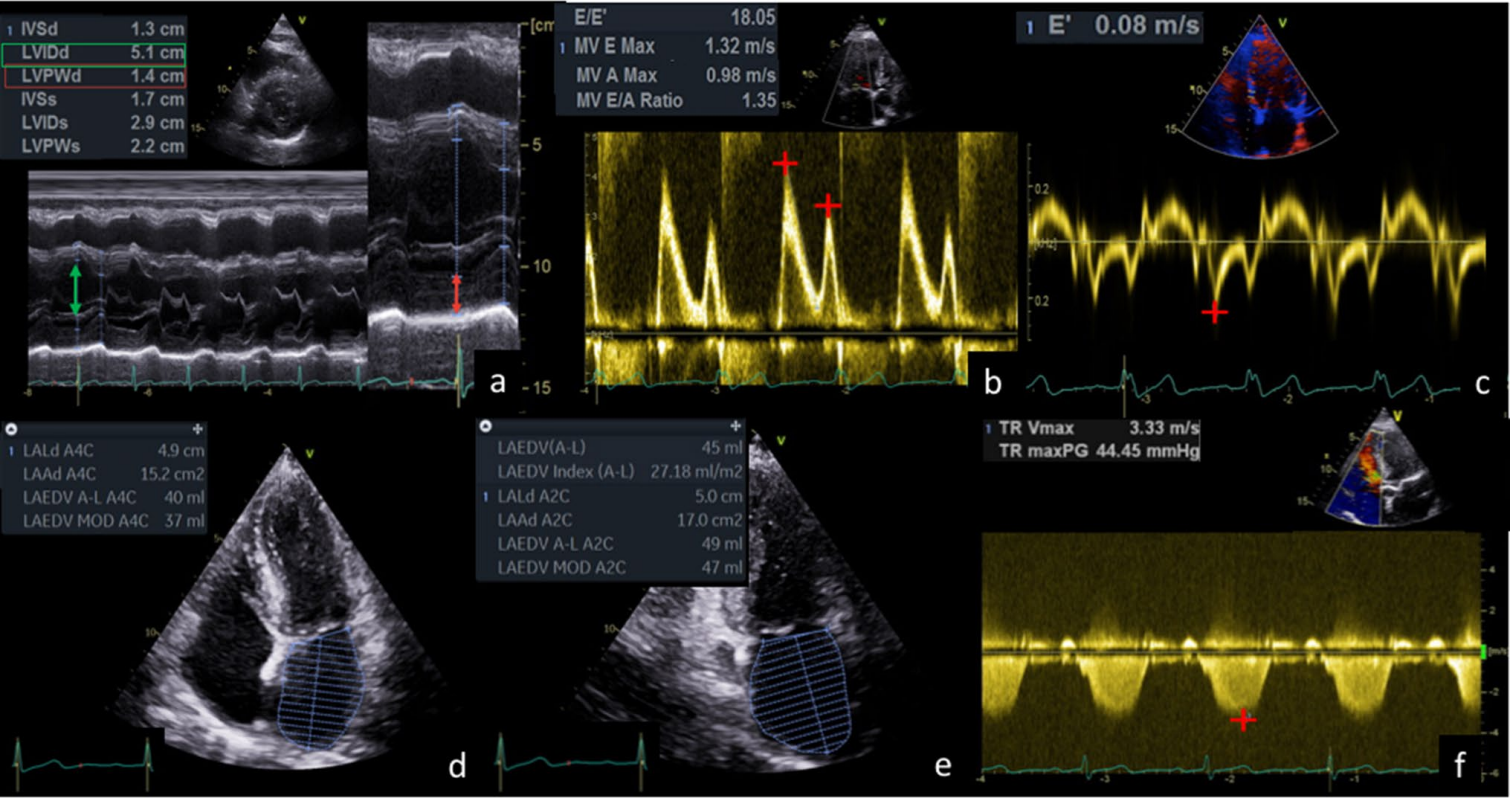
Determination of left ventricular hypertrophy (**a**), diastolic dysfunction (**b**-**f**) and pulmonary arterial hypertension (**f**) by echocardiography. IVSd = diameter of interventricular septum, LVIDd = left ventricular internal dimension at end-diastole, LVPWd = left ventricular posterior wall diameter, LAEDV = left atrial end diastolic volume, TR = tricuspid regurgitation, Vmax = maximum velocity, maxPG = maximum pressure gradient, MV = mitral valve

Relative wall thickness **(**RWT) was calculated by twice of the LV posterior wall diameter (LVPWD) divided by LV end-diastolic diameter (LVEDD) (Fig. 3a) [9]. LV mass (LVM) was calculated by the following equation: LVM (g) = 0.8 × {1.04 × [([LVEDD + diameter of the interventricular septum + LVPWD]^3^–LVEDD^3^)]} + 0.6 and indexed to the BSA (LVM_i_). Normal RWT was defined ≤ 0.42 and normal LVM_i_ was defined ≤ 95 g/m^2^ (female) or ≤ 115 g/m^2^ (male). Using RWT and LVM_i_, LV geometry was categorized in four groups: normal LV geometry (RWT ≤ 0.42 and normal LVM_i_), eccentric LVH (RWT ≤ 0.42 and increased LVM_i_), concentric LV remodelling (RWT > 0.42 and normal LVM_i_) and concentric LVH (RWT > 0.42 and increased LVM_i_) [9].

### Diastolic dysfunction

Transmitral LV inflow was assessed by PW-Doppler placing the sample volume at the tips of the mitral leaflets to measure E-wave (passive filling), A-wave (atrial contraction) and E/A ratio (Fig. 3b). E’ was calculated by averaging the early passive filling velocity determined by tissue Doppler imaging (TDI) placing the sample volume at the basal inferoseptal and lateral mitral annulus (apical 4-chamber view) (Fig. 3c) [8]. Indexed left atrial end-diastolic volume (LAEDV) was measured by LA planimetry in the apical 2- and 4-chamber view at LV end-systole and LAVI > 34 ml/m^2^ was defined as abnormal (Fig. 3d,e) [8].

For patients with sinus rhythm (SR), DD grade 2 was defined by E/A ≤ 0.8 + E > 50 cm/s or E/A > 0.8—< 2 and in presence of 2 out the following 3 parameters (LAVI > 34 ml/m^2^, E/E' > 14 and regurgitation flow of the tricuspid valve > 2.8 m/s), or DD grade 3 when E/A ≥ 2 [22]. In case of atrial fibrillation (AF) DD grade 2 or 3 was defined by E/E’ > 11 and/or LAVI > 34 ml/m^2^ [23]. In all patients with SR 3 cycles were averaged, in patients with AF 5 cycles.

### Pulmonary artery hypertension (PAH)

sPAP was assessed by measuring maximum velocity of tricuspid regurgitation (TR-V_max_) using CW Doppler (Fig. 3f) according to the simplified Bernoulli equation: sPAP = 4 × (TR−V_max_)^2^ adding the estimated central venous pressure [8]. sPAP > 35 mmHg was defined as pathological [24].

### Statistical analysis

All statistical analyses were performed using SPSS Statistics version 24.0 (IBM, Armonk, NY). Continuous variables were expressed as mean value ± standard deviation (SD) and were compared between groups using Student’s t-test. All categorical variables were expressed as numbers with their percentages (%) and compared using chi-squared or Fisher exact test, as appropriate. Kolmogorov–Smirnov test was performed to test normal distribution of the population. Linear regression and Pearson’s r were applied to evaluate association between two linear variables. Data comparisons between more than two groups were performed by one-way Analysis of Variance (ANOVA). A p value < 0.05 was considered to indicate statistical significance.

Intraobserver variability was assessed by repeating all measurements under the same conditions in 20 patients. Further, interobserver variability was assessed by measurements of a second investigator who was unaware of the results of the first examination.

## Results

### Basic echocardiographic parameters and hemodynamics

**Fig. 4:**
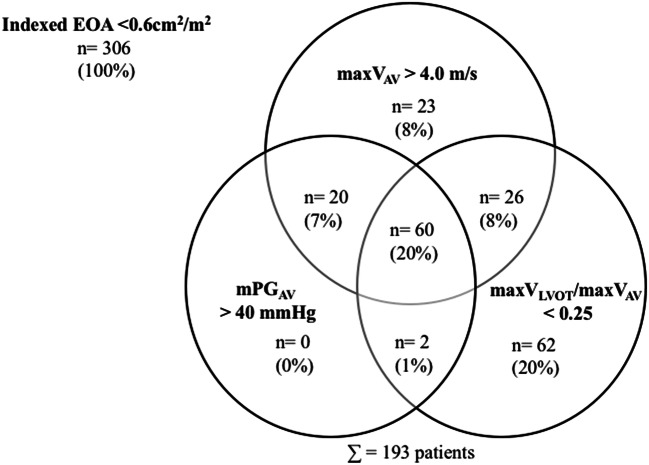
Circle diagram to illustrate the intersections between the presence of maxV_AV_ > 4.0 m/s, mPG_AV_ > 40mmHG, and maxV_LVOT_/maxV_AV_ in patients with “pure” severe AS defined by EOA < 0.6 cm^2^/m^2^ according to current guidelines: EOA < 0.6 cm^2^/m^2^ was documented in 113 “pure” AS patients without either a significant increase of maxV_AV_ or mPG_AV_ or a decrease of maxV_LVOT_/maxV_AV_. maxV_AV_ = peak transvalvular flow velocity; mPG_AV_ = mean transvalvular pressure gradient; maxV_LVOT_/maxV_AV_ = ratio between peak velocity determined at the level of LV outflow tract (maxV_LVOT_) and maxV_AV_

Only 60 (20%) of 306 (mean age 78 ± 9.5 years; females 53%) “pure” severe AS patients defined by EOA according to current recommendations met all guideline criteria for severe AS: maxV_AV_ > 4 m/s, mPG_AV_ > 40mmHG and maxV_LVOT_/maxV_AV_ < 0.25 (Fig. 4). Further, in only 131 patients (43%) an increased mPG_AV_ and/or maxV_AV_ were observed (Fig. 4). Thus, 113 patients (37%) were solely classified as severe AS by EOA due to continuity equation without either a significant increase of maxV_AV_ or mPG_AV_ or a decrease of maxV_LVOT_/maxV_AV_.

**Fig. 5:**
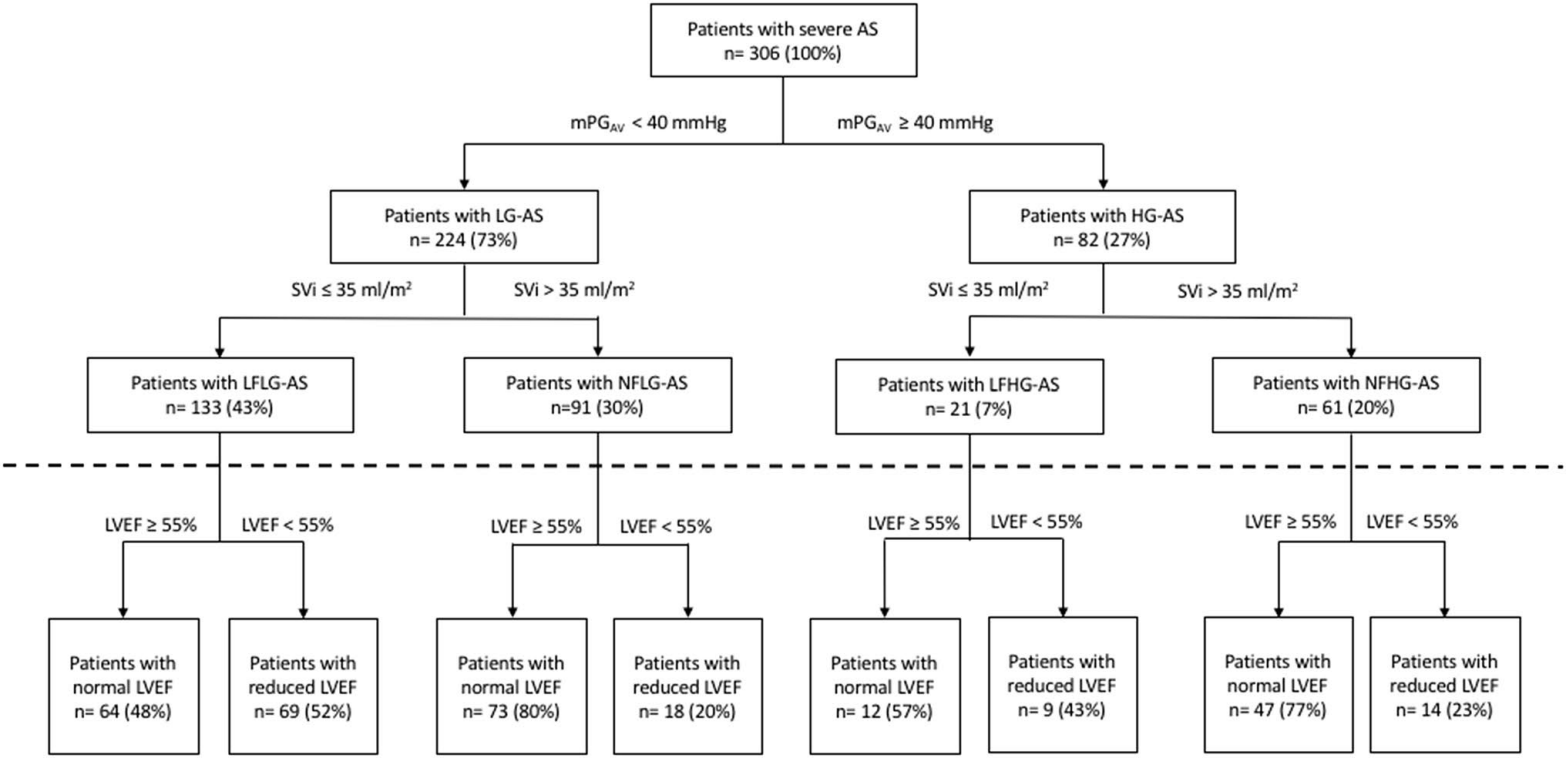
Selection of the study population with respect to mPG_AV_ and SVi. Patients were divided according to the ESC/EACTS guidelines for the management of valvular heart disease (2017). AS = Aortic stenosis; LG = low gradient; HG = high gradient; LFLG = low flow low gradient; NFLG = normal flow low gradient; LFHG = low flow high gradient; NFHG = normal flow high gradient; SV_i_ = stroke volume index; mPG_AV_ = mean pressure gradient of the aortic valve; LV = left ventricle; EF = ejection fraction

224 (73%) patients showed LG-conditions and only 82 (27%) showed HG-conditions (Fig. 5). LF-conditions were observed in 154 (50%) patients, NF-conditions in 152 (50%) patients (Fig. 5). Patients with LF-conditions were older and had more often AF. Particularly LFLG-AS patients showed increased prevalence of comorbidities, e.g. arterial hypertension and diabetes mellitus (Table 1).

**Table 1:** Baseline characteristics

Variables	All Patients (n = 306)	LFLG-AS (n = 133)	NFLG-AS (n = 91)	LFHG-AS (n = 21)	NFHG-AS (n = 61)	p value
Age, years	78.1 ± 9.0	80.0 ± 8.4*⧧	77.9 ± 8.9†⧧	81.1 ± 6.3 ⧧	73.4 ± 11.6	< 0.001
Female, n	161 (53%)	76 (57%)	49 (54%)	9 (43%)	27 (44%)	NS
Atrial fibrillation	112 (37%)	69 (51%)*⧧	19 (21%)†	14 (67%)⧧	10 (13%)	< 0.001
Ischemic stroke/TIA	49 (16%)	27 (20%)	12 (12%)	3 (14%)	7 (10%)	NS
Hypertension	228 (75%)	105 (79%)*	61 (67%)	16 (76%)	46 (75%)	NS
Hyperlipidemia	58 (19%)	25 (19%)	17 (19%)	4 (19%)	12 (20%)	NS
Diabetes mellitus	112 (37%)	58 (44%)*	27 (30%)	7 (33%)	20 (33%)	NS
CAD	97 (32%)	48 (36%)	27 (30%)	5 (24%)	17 (28%)	NS
COPD	29 (9%)	14 (11%)	8 (9%)	2 (10%)	5 (8%)	NS
Chronic kidney disease	101 (33%)	49 (37%)	28 (31%)	6 (29%)	18 (30%)	NS
Vertigo	62 (20%)	28 (21%)	20 (22%)	5 (24%)	9 (15%)	NS
Dyspnea	126 (41%)	56 (42%)	42 (46%)	10 (48%)	18 (30%)	NS
Chest pain	44 (14%)	19 (14%)	11 (12%)	4 (19%)	10 (16%)	NS
Syncope	10 (3%)	5 (4%)	1 (1%)	1 (5%)	3 (5%)	NS
ACE-inhibitor	122 (40%)	48 (36%)	41 (45%)	12 (57%)	21 (34%)	< 0.001
ß-Blocker	170 (56%)	80 (60%)*†	54 (59%)⧧	10 (48%)	26 (43%)	NS
AR-Blocker	77 (25%)	33 (24%)*	17 (19%)	1 (5%)	14 (23%)	< 0.001
Diuretics	159 (52%)	78 (59%)*⧧	39 (43%)†	16 (76%)⧧	26 (43%)	< 0.001
Statins	124 (41%)	53 (40%)	37 (41%)	10 (48%)	24 (39%)	NS
CCB	67 (22%)	24 (18%)*	29 (32%)	3 (14%)	11 (18%)	0.001
Echocardiographic parameters						
EOA, cm^2^	0.74 ± 0.15	0.74 ± 0.16*†	0.82 ± 0.15†⧧	0.52 ± 0.11⧧	0.72 ± 0.15	< 0.001
maxV_AV_, m/s	3.8 ± 0.6	3.2 ± 0.6*†⧧	3.8 ± 0.5†⧧	4.6 ± 0.4⧧	4.9 ± 0.6	< 0.001
mPG_AV_, mmHg	31.7 ± 9.1	21.3 ± 8.9*†⧧	29.0 ± 6.9†⧧	49.8 ± 7.6	52.3 ± 13.2	< 0.001
LVSV (doppler), ml	64.2 ± 11.7	48.6 ± 10.3*†⧧	75.3 ± 11.9†⧧	55.2 ± 11.1⧧	84.7 ± 14.5	< 0.001
LVSV (Doppler) index, ml/m^2^	34.8 ± 5.7	26.1 ± 5.1*†⧧	42.5 ± 6.0†⧧	28.2 ± 4.6⧧	44.6 ± 6.7	< 0.001
LVEDV index, ml/m^2^	55.2 ± 18.6	52.6 ± 21.4⧧	57.4 ± 16.5	52.6 ± 14.4	58.3 ± 17.1	NS
LVESV index, ml/m^2^	25.0 ± 13.3	27.7 ± 17.2*⧧	23.2 ± 10.9	24.1 ± 10.7	22.3 ± 9.5	0.03
LVSV (biplane), ml	57.9 ± 19.4	50.5 ± 19.2*⧧	63.4 ± 18.6	56.4 ± 19.3⧧	66.4 ± 21.1	< 0.001
LVSV index (biplane), ml/m^2^	31.7 ± 14.2	28.2 ± 20.6*⧧	35.5 ± 8.5†	28.5 ± 9.0⧧	34.9 ± 10.2	0.001
Cardiac output (L/min)	4.7 ± 1.1	3.8 ± 1.1*†⧧	5.1 ± 1.0†⧧	4.3 ± 1.0⧧	6.2 ± 1.4	< 0.001
Cardiac index (L/min/m^2^)	2.5 ± 0.6	2.0 ± 0.6*†⧧	2.9 ± 0.5†⧧	2.2 ± 0.4⧧	3.3 ± 0.7	< 0.001
LVEF, %	55.8 ± 11.0	49.8 ± 13.4*†⧧	60.5 ± 8.2†⧧	55.3 ± 11.8⧧	62.1 ± 9.8	< 0.001

*LFLG* low flow low gradient, *NFLG* normal flow low gradient, *LFHG* low flow high gradient, *NFHG* normal flow high gradient, *BMI* body-mass-index, *CAD* coronary artery disease, *COPD* chronic obstructive lung disease, *ACE* angiotensin converting enzyme, *AR* aldosterone receptor, *CCB* calcium channel blocker, *EOA* effective orifice area, *LV* left ventricle, *SV* stroke volume, *ESV* end-systolic volume, *EDV* end-diastolic volume, *EF* ejection fraction, *maxV*_*AV*_ peak transvalvular flow velocity of aortic valve, *mPG*_*AV*_ mean transvalvular pressure gradient of aortic valve

*Significant difference (p < 0.05) with normal-flow low-gradient (NFLG) group. †significant difference with low flow high gradient (LFHG) group. ⧧ significant difference with normal flow high gradient (NFHG) group

Normal LVEF was observed in 196 (64%) “pure” AS patients, reduced LVEF in 110 (36%) patients. The proportion of normal LVEF was significantly higher in NFHG-AS patients in comparison to other AS subgroups (Table 1 and Fig. 5). LVSV showed significant differences between SV_LV-Doppler_ and SV_LV-bipl_ in patients with NF-AS (NFLG-AS: 75.3 ± 11.9 vs. 63.4 ± 18.6 ml/m^2^; NFHG-AS: 84.7 ± 14.5 vs. 66.4 ± 21.1 ml/m^2^; p < 0.001) as well as between indexed SV_LV-Doppler_ and indexed SV_LV-bipl_ in patients with NF-AS (NFLG-AS: 42.5 ± 6.0 vs. 35.5 ± 8.5 ml/m^2^; NFHG-AS: 44.6 ± 6.7 vs. 34.9 ± 10.2 ml/m^2^; p < 0.001), whereas no differences were observed in LF-AS subgroups (Table 1).

### Left ventricular geometry

Most patients showed concentric LVH (n = 243, 79%) irrespectively of AS subtypes with an increased presence of LVH in NF-AS compared to LF-AS patients (86% vs. 73%; p = 0.005). Normal LV geometry was only observed in 7 LG-AS patients (Table 2).

**Table 2:** Parameter of LV geometry

Variables	All patients (n = 306)	LFLG-AS (n = 133)	NFLG-AS (n = 91)	LFHG-AS (n = 21)	NFHG-AS (n = 61)	p value
RWT > 0.42	285 (93%)	121 (91%)⧧	84 (92%)⧧	19 (90%)⧧	61 (100%)	0.036
LVMI > 115 g/m^2^ in men and > 95 g/m^2^ in women	256 (84%)	105 (79%)⧧	78 (86%)	17 (81%)	56 (92%)	NS
Normal geometry	7 (2%)	4 (3%)	3 (3%)	0 (0%)	0 (0%)	NS
Eccentric hypertrophy	14 (5%)	8 (6%)†	4 (4%)	2 (10%)⧧	0 (0%)	NS
Concentric remodeling	42 (13%)	23 (17%)	10 (11%)	4 (19%)	5 (8%)	NS
Concentric hypertrophy	243 (79%)	98 (74%)⧧	74 (81%)	15 (71%)⧧	56 (92%)	0.014

*LFLG* low flow low gradient, *NFLG* normal flow low gradient, *LFHG* low flow high gradient, *NFHG* normal flow high gradient, *RWT* relative wall thickness, *LVMI* indexed left ventricular mass

*Significant difference (p < 0.05) with normal flow low gradient (NFLG) group. † significant difference with low flow high gradient (LFHG) group. ⧧ significant difference with normal flow high gradient (NFHG) group

### Diastolic dysfunction

**Fig. 6:**
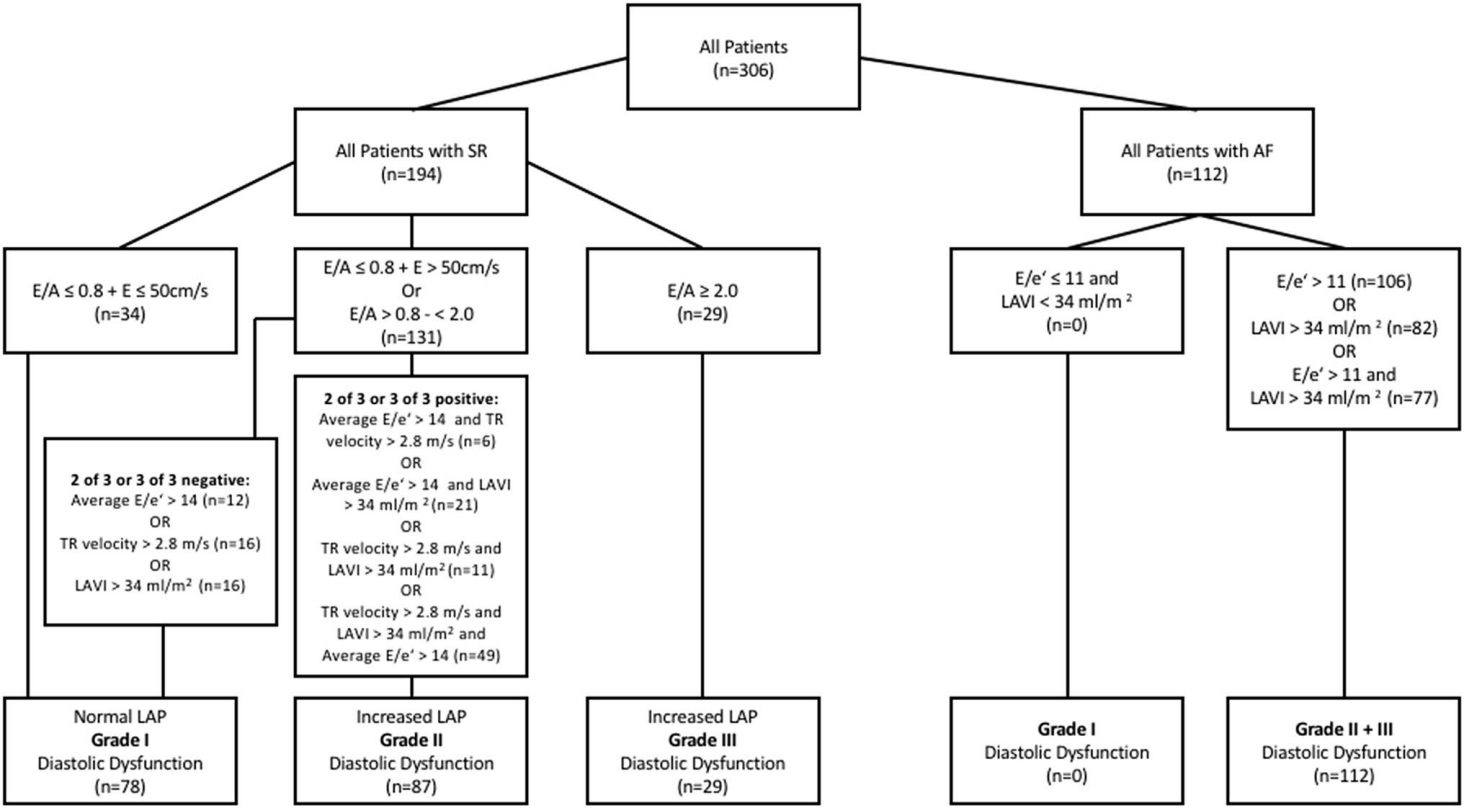
Selection of the study population with respect to diastolic dysfunction. SR = Sinus rhythm, AF = Atrial fibrillation; LAVI = indexed left atrial volume; TR = Tricuspid regurgitation; LAP = Left atrial pressure

Increased E/E’ (> 14 with SR / > 11 with AF) was observed in 212 (69%) “pure” AS patients. LA dilatation was observed in 182 (59%) “pure” AS patients with significant differences between AS subtypes (Table 3). Increased LV filling pressure and at least DD grade 2 were observed among 226 (75%) patients, without significant differences among AS subtypes (Table 3). Among LV filling velocities, A-wave velocities were lower among LF-AS in comparison to NF-AS patients (p < 0.001) whereas no differences were observed for E-wave velocities. In “pure” severe AS patients with SR (n = 194, 60%) 116 patients showed DD grade 2 or 3. In “pure” severe AS patients with AF (n = 112, 100%) all patients showed DD grade 2 or 3 (Fig. 6).

**Table 3:** Parameters of diastolic function in AS subgroups

Variables	All Patients (n = 306)	LFLG (n = 133)	NFLG (n = 91)	LFHG (n = 21)	NFHG (n = 61)	p value
E-wave velocity, m/s	1.0 ± 0.36	1.05 ± 0.36	0.99 ± 0.35	0.99 ± 0.31	0.98 ± 0.37	NS
A-wave velocity, m/s	0.89 ± 0.39	0.75 ± 0.45*⧧	1.03 ± 0.35†	0.78 ± 0.34⧧	1.01 ± 0.33	< 0.001
E/A ratio	1.38 ± 1.18	1.85 ± 1.67*†⧧	1.06 ± 0.89	0.96 ± 0.46	1.00 ± 0.78	< 0.001
E', m/s	0.06 ± 0.02	0.06 ± 0.02	0.06 ± 0.02	0.06 ± 0.02	0.06 ± 0.02	NS
E/E' ratio	19.6 ± 9.6	21.3 ± 11.4*†⧧	18.8 ± 8.7	17.6 ± 5.1	18.0 ± 8.4	NS
E/ E' > 14 (11*)	212 (69%)	96 (72%)	62 (68%)	15 (71%)	39 (64%)	NS
LAVI, ml/m^2^	40.8 ± 15.3	41.2 ± 16.7	39.1 ± 12.0	42.3 ± 14.1	41.7 ± 17.7	NS
LAVI > 34 ml/m^2^	182 (59%)	62 (47%)*†	69 (73%)†⧧	19 (90%)⧧	32 (52%)	< 0.001
sPAP, mmHg	47.2 ± 14.9	49.3 ± 18.4*	42.5 ± 10.8†⧧	51.4 ± 13.2	48.3 ± 13.9	0.002
sPAP > 35 mmHg	245 (80%)	100 (75%)†	71 (78%)†	21 (100%)	53 (87%)	0.014
Increased LAP and Grade 2 or 3 diastolic dysfunction	228 (75%)	103 (76%)	63 (70%)	18 (81%)	44 (75%)	NS

*LFLG* low flow low gradient, *NFLG* normal flow low gradient, *LFHG* low flow high gradient, *NFHG* normal flow high gradient, *LAVI* indexed left atrial volume, *LAP* left atrial pressure, *TR* tricuspid valve regurgitation

*Significant difference (p < 0.05) with normal flow low gradient (NFLG) group. ^†^Significant difference with low flow high gradient (LFHG) group

^⧧^significant difference with normal flow high gradient (NFHG) group

### Pulmonary artery hypertension

sPAP was > 35 mmHg in 245 (80%) “pure” severe AS patients. It was significantly higher in HG-AS (90%) vs. LG-AS (76%) patients (p = 0.007). No differences were observed according to flow conditions (p = 0.508) (Table 3).

### Prevalence of secondary cardiac alterations

The presence of LVH, DD grade 2 or 3, and PAH as well as the incidence of their combination are shown in Table 2 and 3. One of these secondary alterations were observed in 51 (17%), two in 90 (29%) and all three in 165 (54%) of “pure” AS patients. LG-AS patients had the lowest presence of all three secondary alterations–especially LFLG-AS with normal LVEF and NFLG-AS with reduced LVEF. Patients, in whom all three secondary cardiac alterations were present, showed higher maxV_AV_ (3.9 ± 0.9 vs 3.6 ± 0.8, p = 0.002) and higher mPG_AV_ (34.0 ± 16.6 vs 29.3 ± 14.1, p = 0.009).

### Symptoms, comorbidities and medication

According to clinical reports 173 (57%) patients with “pure” severe AS were classified as symptomatic, although also unspecific symptoms e.g. chest pain, vertigo, reduced resilience or performance, clinical signs of heart failure and/or syncope at rest have been accepted. In 76 (44%) of 173 symptomatic patients a causal relationship between symptoms and severe AS (angina without coronary artery disease and history of hypertension; stress-induced syncope) was highly likely. Unspecific dyspnea was the leading symptom in all symptomatic patients followed by other unspecific symptoms like vertigo and chest pain. The presence of dyspnea (p = 0.023) and chest pain (p = 0.049) were higher in patients with three secondary cardiac alterations in comparison to patients with at least one or two. Syncope was rare among all AS subgroups. The distribution of symptoms did not significantly differ between AS subgroups (Table 1). The presence of AF (p < 0.001) and chronic kidney disease (p = 0.029) was higher in patients with three secondary cardiac alterations than in patients with one or two. Between AS subgroups, dosages of ß-blockers and statins did not significantly differ, whereas significant differences were observed for ACE-Inhibitors, AR-Blocker, Diuretics, and Calcium Channel (Table 1).

### Treatment of patients with severe AS

**Fig. 7:**
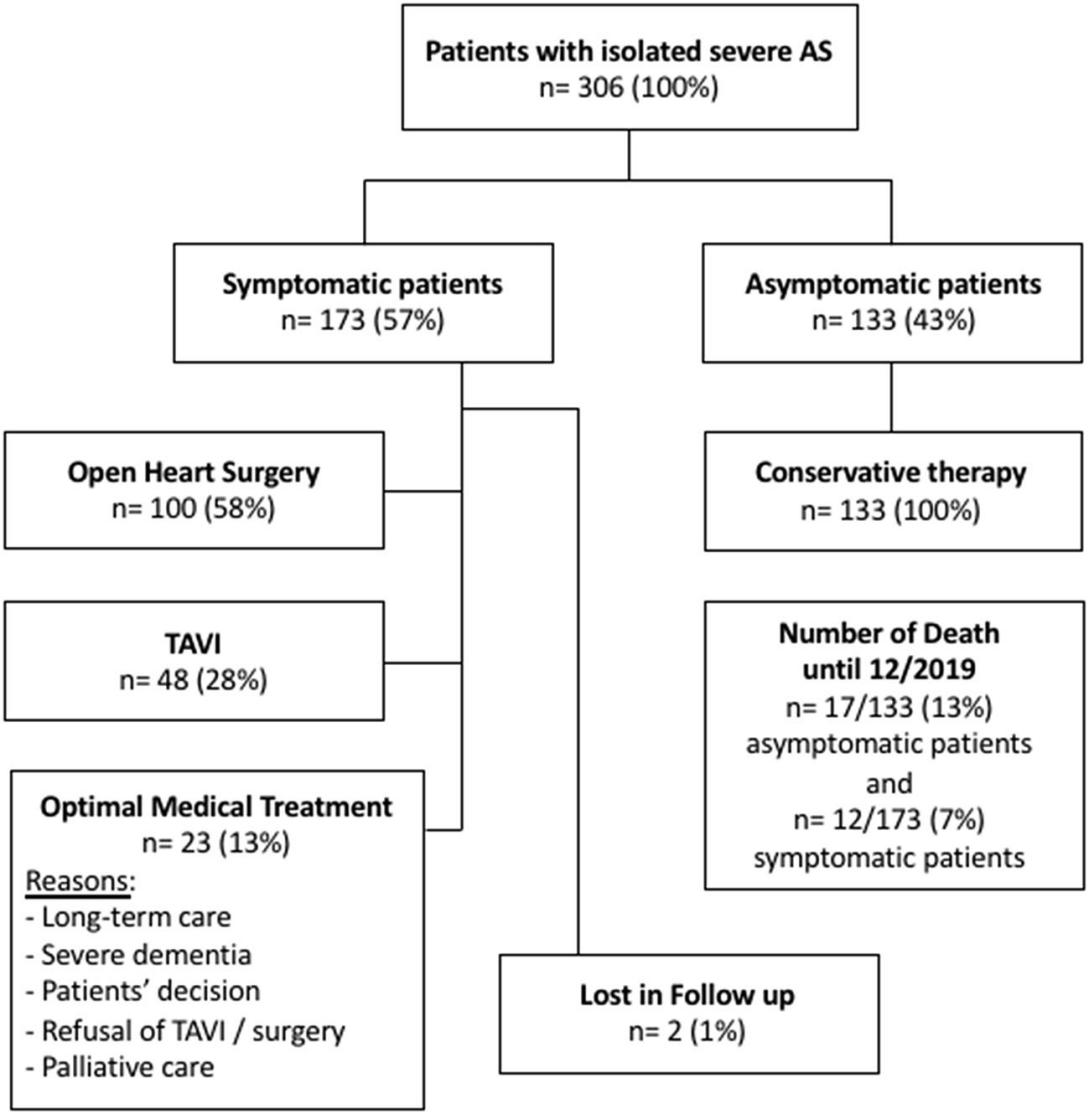
Retrospective data analysis in patients with “pure” severe AS until december 2019. AS = Aortic stenosis; TAVI = transcatheter aortic valve implantation

Symptomatic AS patients (n = 173, 57%) were treated by surgery (n = 100, 58%), TAVI (n = 48, 28%) and OMT (n = 23, 13%), 2 AS patients were lost to follow-up. Symptomatic patients were treated by OMT due to different reasons, e.g. need for long-term care, severe dementia, patient’s decision, refusal of TAVI or surgery, cancer in palliative care (Fig. 7).

All asymptomatic patients (n = 133, 43%) were treated by OMT. Until December 2019 12 (7%) symptomatic and 17 (13%) asymptomatic patients died (Fig. 7).

### Inter- and intraobserver variabilities

Inter- and intraobserver variabilities of all echocardiographic measurements were 9.3% and 7.5%, respectively.

## Discussion

The main findings of the present study are:

1. *If “pure” severe AS is assessed by EOA according to current echocardiographic recommendations, 54% of AS patients presented LVH, DD and PAH in combination. Thus, the hypothesis of the assumed presence of the accepted pathophysiological consequences of severe AS was not confirmed.*
2. *LVH, DD and PAH were significantly more often present in HG-AS than in LG-AS. Thus, the hypothesis, that AS subgroups might have no influence on cardiac remodeling, was not confirmed.*
3. *According to current guideline criteria (EOA indexed* < *0.6cm*^*2*^*/m*^*2*^*) an astonishing high proportion of LG-AS was observed in “pure” severe AS patients.*
4. *Symptoms cannot be used as a convincing criterium to characterize AS severity in the elderlies. Most of the symptoms are unspecific and a causal relationship cannot be proven.*
5. *Methodologically, no differences were observed for LVSV- and SVi-assessment by Doppler echocardiography in comparison to LV planimetry in LF-AS. In contrast, LVSV- and SVi determined by Doppler echocardiography were significantly higher in NF-AS.*

### Characterization of the study population

The exceptionality of the present study is that patients with concomitant valvular heart diseases which might have additional effects on cardiac morphology and function are excluded. Thus, pre- and transvalvular Doppler parameters are not additionally influenced by concomitant heart valve diseases. To our knowledge this is the first comparably pre-selected study about a cohort of “pure” severe AS patients defined by EOA according to current guideline criteria. In particular mild AR was excluded because (1) AR might be underestimated by semi-quantitative evaluation in patients with AS and small cavities, and (2) even mild AR might lead to overestimation of flow conditions by determination of a higher SV_LV-Doppler_. Patients with mitral and tricuspid regurgitation were also excluded, because LV and RV volume overload would have a significant impact on LV and RV geometry and pulmonary vascular resistance. In addition, AS patients with indexed EOA > 0.6cm^2^/m^2^ were excluded to avoid non-severe AS due to hyperdynamic state.

The importance of a detailed characterization of the echocardiographic inclusion criteria is underlined by the differences between SV_LV-Doppler_ and SV_LV-bipl_ in NF-AS. In the present study SV_LV-Doppler_ was about 20% higher than SV_LV-bipl_ in NF-AS patients. Obviously, there are methodological aspects influencing VTI_LVOT_ in NF-AS patients–presumably because the PW-Doppler sample volume is positioned in region of the pre-stenotic proximal convergence zones or blood flow velocities, which are affected by turbulences due to subvalvular septal bulging–leading to overestimation of flow conditions. To our knowledge either SV_LV-Doppler_ or SV_LV-bipl_, but not simultaneously both parameters have been assessed in previous studies, whereby no significant differences have been observed between the different flow conditions in severe AS patients [22, 25–32]. However, methodological aspects cannot explain the surprising high proportion of LG-AS patients in the present study. It can be assumed that every amount of AR might have a significant influence on SV_LV-Doppler_, which will lead to an overestimation of flow conditions. Thus, the relevance of AR in severe AS should be analyzed quantitatively in future trials.

In contrast to previous studies AS patients with normal as well as reduced LVEF were included, because the continuity equation for the assessment of EOA is used regardless of an impairment of LV systolic function according to current guidelines. It has to be considered that about 39% of LG-AS patients had reduced LVEF (presumably due to concomitant coronary artery and hypertensive heart disease) which might contribute to the high proportion of LG-AS patients in the present study. Especially the number of LFLG-AS was fourfold higher than previously reported [22, 33]. This might be explained by: (1) the inclusion of symptomatic as well as asymptomatic AS patients and (2) the increased age of the present population in contrast to the study population of Lancellotti et al. reporting only about asymptomatic AS in younger patients with normal LVEF [22].

### Secondary cardiac alterations in “pure” severe AS – concentric LVH, DD, PAH

According to pathophysiological adaptations due to AV narrowing, it can generally be assumed that all secondary cardiac alterations (LVH, DD and PAH) might be present in hemodynamically relevant chronic AS. Thus, the prevalence of these alterations is expected to be higher in these patients than in the normal age-matched population. However, the present data cannot support these pathophysiological sequelae, because only 54% of “pure” severe AS patients showed all secondary cardiac alterations. In principle, the following explanations are possible: (1) it is not fundamentally necessary, that severe AS is accompanied with all secondary cardiac alterations, (2) it is possible, that the definition of severe AS according to current guideline criteria by EOA might include also moderate or hemodynamically non-relevant AS or (3) a combination of both.

Thus, if non-relevant AS would be classified as severe AS by continuity equation, the incidence of combined secondary cardiac alterations might be supportive to diagnose AS severity with a higher probability—especially in “pure” severe AS. The proportion of combined LVH, DD and PAH was increased in asymptomatic and symptomatic “pure” AS patients defined by EOA < 0.6 cm^2^/m^2^ with maxV_AV_ > 4 m/s and mPG_AV_ > 40 mmHg in comparison to LG-AS conceivably underlining misinterpretation of AS severity – especially in LG-AS patients.

In previous studies LVH, DD and PAH have also been associated with the patients’ outcome [12, 33–37]. Further, LVH implicates a poorer prognosis and higher mortality after AV replacement [11]. In the present study, the highest E/E’-values were found in LFLG-AS, which may contribute to the poorer outcome of these patients [22, 25, 38]. The presence of DD in AS patients is already known and depends on flow conditions [16]. The presence of PAH was in line with previous studies [3, 39, 40]. However, significantly higher values were observed in LF-AS compared to NF-AS (Table 3). An increased pre-operative sPAP showed an increased mortality and decreased long term-survival in comparison to patients with normal sPAP prior to surgery [39, 40].

### Correlation of symptoms to AS severity

Cardinal symptoms of severe AS are stress-induced angina, dyspnea and/or syncope [9, 10]. However, the precise prevalence of symptoms in patients with severe AS is still not known, because the causal relationship between symptoms and AS severity is hard to define. However, most of the symptoms are unspecific and cannot conclusively be associated with AS in the elderlies. Thus, it is not surprising that the occurrence of symptoms is usually not suitable for characterization of AS severity.

In the present study LF-AS patients tend to be older in comparison to NF-AS. Further, AF tend to be observed more often in LF-AS. The correlation between AF and increased LA pressure as well as age is already known and in agreement with previous studies [41, 42].

### Further Classification of AS

Generaux et al. presented an echocardiographic classification regarding the outcome of patients with severe AS based on an extent of structural cardiac changes (abnormalities of LV, RV, LA and mitral or tricuspid valve). Concomitant valvular heart diseases obviously have had a significant impact on the patients’ outcome [43, 44]. The patients` cohort of the present study cannot be compared to these data, because all further relevant valvular heart diseases have been excluded.

### Limitations

The selection of “pure” severe AS patients defined by EOA assessment explains the relatively small number of AS patients in the present study. However, these highly selected severe AS patients without concomitant valvular heart diseases highlight the exceptionality of the present cohort. This retrospective cross-sectional study does obviously not allow conclusions about the patients’ outcome and the development of LVH, DD and PAH with disease progression. The follow-up until 12/2019 documented a high percentage of AV treatment in symptomatic severe AS patients. In addition, the prevalence and the incidence rate of secondary cardiac alterations in AS patients with concomitant valvular heart diseases have to be analyzed in further trials. In the present study assessment of LV remodeling and AS severity by cardiac magnetic resonance (CMR) could not be performed due to missing CMR data sets. Probably, the small sample size of LFHG-AS has an impact of the statistical significance between AS subgroups.

## Conclusions

The echocardiographic characterization of “pure” severe AS based on EOA by the continuity equation might implicate diagnostic incongruencies. In patients with “pure” severe AS according to current guideline criteria the presence of combined LVH, DD and PAH as accepted pathophysiological sequelae cannot be confirmed. Probably, the detection of these secondary cardiac alterations might improve the diagnostic algorithm to avoid overestimation of AS severity. The high proportion of LG-AS in this preselected cohort highlights the importance of concomitant valvular diseases for characterizing flow conditions in AS patients. In addition, flow conditions might be overestimated by SV_LV-Doppler_ assessment in presence of (even mild) AR. Thus, these findings might have implications on future echocardiographic AS classification. The present study sets the stage for follow-up studies to determine the prognostic value of secondary cardiac alterations in “pure” severe AS.
